# Intravenous pharmacokinetics, oral bioavailability, dose proportionality and in situ permeability of anti-malarial lumefantrine in rats

**DOI:** 10.1186/1475-2875-10-293

**Published:** 2011-10-10

**Authors:** Wahajuddin, Sheelendra P Singh, Kanumuri SR Raju, Asad Nafis, Sunil K Puri, Girish K Jain

**Affiliations:** 1 Pharmacokinetics and Metabolism Division, CSIR-Central Drug Research Institute, Lucknow-226001, Uttar Pradesh, India; 2 Department of Pharmaceutics, National Institute of Pharmaceutical Education and Research, Rae Bareli, India; 3 Parasitology Division, CSIR-Central Drug Research Institute, Lucknow-226001, Uttar Pradesh, India

## Abstract

**Background:**

Despite the wide spread use of lumefantrine, there is no study reporting the detailed preclinical pharmacokinetics of lumefantrine. For the development of newer anti-malarial combination(s) and selection of better partner drugs, it is long felt need to understand the detailed preclinical pharmacokinetics of lumefantrine in preclinical experimental animal species. The focus of present study is to report bioavailability, pharmacokinetics, dose linearity and permeability of lumefantrine in rats.

**Methods:**

A single dose of 10, 20 or 40 mg/kg of lumefantrine was given orally to male rats (N = 5 per dose level) to evaluate dose proportionality. In another study, a single intravenous bolus dose of lumefantrine was given to rats (N = 4) at 0.5 mg/kg dose following administration through the lateral tail vein in order to obtain the absolute oral bioavailability and clearance parameters. Blood samples were drawn at predetermined intervals and the concentration of lumefantrine and its metabolite desbutyl-lumefantrine in plasma were determined by partially validated LC-MS/MS method. *In-situ *permeability study was carried in anaesthetized rats. The concentration of lumefantrine in permeability samples was determined using RP-HPLC.

**Results:**

For nominal doses increasing in a 1:2:4 proportion, the C_max _and AUC_0-∞ _values increased in the proportions of 1:0.6:1.5 and 1:0.8:1.8, respectively. For lumefantrine nominal doses increasing in a 1:2:4 proportion, the C_max _and the AUC_0-t _values for desbutyl-lumefantrine increased in the proportions of 1:1.45:2.57 and 1:1.08:1.87, respectively. After intravenous administration the clearance (Cl) and volume of distribution (Vd) of lumefantrine in rats were 0.03 (± 0.02) L/h/kg and 2.40 (± 0.67) L/kg, respectively. Absolute oral bioavailability of lumefantrine across the tested doses ranged between 4.97% and 11.98%. Lumefantrine showed high permeability (4.37 × 10^-5 ^cm/s) in permeability study.

**Conclusions:**

The pharmacokinetic parameters of lumefantrine and its metabolite desbutyl-lumefantrine were successfully determined in rats for the first time. Lumefantrine displayed similar pharmacokinetics in the rat as in humans, with multiphasic disposition, low clearance, and a large volume of distribution resulting in a long terminal elimination half-life. The absolute oral bioavailability of lumefantrine was found to be dose dependent. Lumefantrine displayed high permeability in the *in-situ *permeability study.

## Background

According to the World Health Organization (WHO), there were in 2008 an estimated 247 million malaria cases among more than 3 billion people at risk, causing nearly one million deaths (even much more according to other estimates), mostly of children under 5 years and pregnant women [1]. The burden of malaria disease continues to increase as the countries in which it is endemic face the risk of widespread resistance of the parasite to conventional anti-malarial drugs and increasing resistance of the vector to insecticide. Artemether/lumefantrine (AL; Coartem^®^) is an artemisinin-based combination therapy (ACT) that offers PCR-corrected 28-day cure rates of > 95% [2-9], if given in a six-dose regimen. AL meets the WHO pre-qualification criteria for efficacy, safety and quality and is the only ACT that has been approved by ICH stringent regulatory authorities [10].

Despite the potency of artemether, 100-100,000 residual parasites remain when the drug is used alone for a three-day treatment course, and as a result up to 10% of patients experience recrudescence [11,12]. It was recognized that combination treatment, which eliminated the final parasites, would be advantageous. Lumefantrine, the other active constituent of AL, acts over a longer period to eliminate the residual 100-100,000 parasites that remain after artemether is cleared from the body and thus minimizes the risk of recrudescence. Artemether and lumefantrine have different modes of action and act at different points in the parasite life cycle [13,14]. Artemisinin derivatives, such as artemether, have multiple mechanisms of action, including interference with parasite transport proteins, disruption of parasite mitochondrial function, modulation of host immune function and inhibition of angiogenesis [15]; Whereas, lumefantrine prevents the detoxification of haem, such that toxic haem and free radicals induce parasite death [13]. Additionally, the differing pharmacokinetics of the two agents offers an advantage for combination therapy. Furthermore, *in vitro*, artemether and lumefantrine have shown synergistic action against *Plasmodium falciparum *under in vitro conditions [16].

The anti-malarial agent lumefantrine, which was originally synthesized by the Academy of Military Medical Sciences in Beijing [17], was identified by researchers at the Academy as a promising agent for combination with artemisinin. Lumefantrine, 2-(dibutylamino)-1-[(9E)-2,7-dichloro-9-[(4-chlorophenyl) methylidene] fluoren-4-yl] ethanol is an arylamino alcohol [14]. Its molecular weight is 528.939 g/mol. It is a lipophillic compound with low intrinsic clearance and erratic oral variability and therapeutic levels are more reliably achieved by co-administration with a fatty meal [14,18-22]. Lumefantrine is eliminated very slowly with a terminal half-life of 2-3 days in healthy volunteers and 4-6 days in patients with falciparum malaria [14,18,23,24]. Its plasma protein binding is almost 100% [25]. Lumefantrine is predominantly, metabolized by cytochrome P450 3A4 (CYP3A4), to desbutyl lumefantrine (DLF). The in vitro antiparasitic effect of desbutyl- lumefantrine is 5 to 8 fold higher than lumefantrine [26].

Recently, Wong *et al *[27] reported that DBL has potential as an anti-malarial drug in its own right. Its in vitro potency relative to that of the parent compound (lumefantrine), its synergy with dihydroartemisinin and the positive relationship between day 7 plasma concentrations and adequate clinical and parasitological response (ACPR) suggest that it could be a useful alternative to lumefantrine as a part of artemisinin-based combination therapy (ACT).

The limitation of artemether-lumefantrine combination is the side effects associated with artemether, i.e hearing impairment, its high cost, and its variable absorption and the strong food effect on the pharmacokinetics of lumefantrine. Development of newer anti-malarial combinations require detailed preclinical pharmacokinetic assessment of combination partner drugs separately as well as in combination for better understanding of their efficacy, toxicity and safety profile before going in to clinical studies. Preclinical pharmacokinetic information is also very useful in dose/dosage regimen selection of combination partner drugs for clinical assessment. For the development of newer anti-malarial combination(s) and selection of better partner drugs, it is long felt need to understand the detailed preclinical pharmacokinetics of existing combination drugs (viz. lumefantrine, artemether etc.) in preclinical experimental animal species.

All the studies reporting pharmacokinetics of lumefantrine dealt with clinical data. Despite the wide spread clinical use of lumefantrine, there is no study reporting the detailed preclinical pharmacokinetics. However, the preclinical pharmacokinetics of artemether in rats has been reported very recently [28]. The focus of present study is to report bioavailability, pharmacokinetics, dose linearity and permeability of lumefantrine. The presence of preclinical pharmacokinetic data in public domain will be of immense help in making informed decisions while selecting the better partner drug(s) for newer combination(s).

## Methods

### Chemicals and reagents

Lumefantrine, desbutyl-lumefantrine and halofantrine (IS) were a generous gift from Ipca Laboratories Ltd. (Mumbai, India). Phenol red and HPLC grade acetonitrile were purchased from Sisco Research Laboratories (SRL) Pvt. Limited (Mumbai, India). HPLC grade n-hexane was obtained from E Merck Limited (Mumbai, India). HPLC grade methanol was purchased from Thomas Baker Pvt. Limited (Mumbai, India). Ammonium acetate, ethanol and glacial acetic acid (GAA) AR were purchased from E Merck Limited (Mumbai, India). Potassium dihydrogen orthophosphate was purchased from New India Chemical Enterprises (Cochin, India). Polyethylene glycol (PEG400) and Carboxy methyl cellulose (CMC) were purchased from Sigma Aldrich Ltd (St Louis, USA). Dimethylformamide was purchased from Thomas Baker (chemicals) Pvt. Limited (Mumbai, India). Urethane was purchased from Thermo Fisher Scientific India Pvt. Ltd. (Mumbai, India). Ultra pure water was obtained from a Sartorious Arium 611 system. Heparin sodium injection I.P. (1000 IU/mL, Biologicals E. Limited, Hyderabad, India) was purchased from local pharmacy. Blank, drug free plasma samples were collected from adult, healthy male Sprague-Dawley (SD) rats at the Division of Laboratory Animals (DOLA) of Central Drug Research Institute (Lucknow, India). Plasma was obtained by centrifuging the heparinized blood (25 IU/mL) at 2000 × g for 10 min at 20°C. Prior approval from the Institutional Animal Ethics Committee (IAEC) was sought for maintenance, experimental studies, euthanasia and disposal of carcass of animals.

### Animals

Young, adult male SD rats, weighing 200-220 g, were procured from the National Laboratory Animal Center, CDRI (Lucknow, India). Rats were housed in well ventilated cages at room temperature (24 ± 2°C) and 40-60% relative humidity while on a regular 12 h light-dark cycle. The animals were acclimatized for a minimum period of three days prior to the experiment. Approval from the Institutional Animal Ethics Committee was sought and the study protocols were approved before the commencement of the studies.

### *In-situ *permeability studies

Single-pass intestinal perfusion studies in rats were performed using established methods adapted from the literature [29]. Briefly, male SD rats were fasted overnight for 12 to 16 h with free access to water and anaesthetized using an intra-peritoneal injection of urethane (1 g/kg) and placed on a heated pad to keep normal body temperature. Upon verification of the loss of pain reflex, a midline longitudinal abdominal incision was made, and the lumen of the jejunum (10 cm) was flushed with 10 ml of saline pre-warmed to 37°C. The proximal end of the lumen was catheterized with an inlet polypropylene tube, which was connected to a perfusion pump. The distal end of the jejunum was also catheterized with an outlet polypropylene tube to collect intestinal effluent. Care was taken to handle the small intestine gently and to minimize the surgery in order to maintain an intact blood supply. The entire excised area was covered with an absorbable cotton pad soaked in warmed normal saline. After allowing 30 min to reach steady-state outlet concentrations, outlet perfusate samples were collected every 15 min for 120 min perfusion period. Phenol red was used as a marker of osmosis/zero permeability. At the end, the length of segment was measured without stretching and finally the animal was euthanized. Samples were stored at -20°C until analysis.

### HPLC analysis of *In-situ *permeability samples

The concentration of lumefantrine and phenol red in permeability samples was determined by high-performance liquid chromatography (HPLC) coupled with PDA detector. Chromatographic separation was performed on a Supelco Discovery C18 column (4.6 × 150 mm, 5.0 μm). Mobile phases were duly filtered through 0.22 μm Millipore filter (Billerica, USA) and degassed ultrasonically for 15 min and then were pumped in gradient mode. The detail of the gradient program is given in Table 1. The lumefantrine and phenol red were detected at the wavelength of 235 and 420 nm, respectively.

**Table 1:** HPLC gradient used for the determination of lumefantrine and phenol red in *in-situ *permeability samples

Time (Minute)	Solvent A	Solvent B	Flow rate (mL/min)
0 - 4	65	35	1
4 - 12	30	70	
12 - 17	65	35	

**Solvent A: **KH_2_PO_4 _buffer (10 mM, pH-3)

**Solvent B: **Methanol

### Permeability data analysis

The single pass intestinal perfusion is based on reaching steady state with respect to the diffusion of compound across intestine. Steady state is confirmed by plotting the ratio of the outlet to inlet concentrations (corrected for water transport) versus time. The outlet concentrations were corrected by multiplying the inlet concentration with [phenol red]*_in _*/[phenol red]*_out_*. Permeability calculations across rat jejunum *(P_eff_) *were performed from intestinal perfusate samples collected over 30-120 min (steady state).

[phenol red]*_in _*and [phenol red]*_out _*are the inlet and outlet concentrations of the water flux marker phenol red. The effective permeability coefficient (*P_eff_*) and drug absorption rate constant (*K_a_*) were calculated using the following equations:

(1) $$P_{eff}=\frac{-Q_{in}\;ln\left[\frac{C_{out}}{C_{in}}\right]}{2\pi \text{r}l}$$

(2) $$K_{a}=\left[1-\frac{C_{out}}{C_{in}}\right]\times \frac{Q_{in}}{\pi r^{2}l}$$

Where, *C_out _*is the corrected concentration of the permeant in the exiting perfusate; *C_in _*is the concentration of the permeant in entering perfusate; *Q_in _*is the flow rate of entering perfusate (0.2 mL/min); *r *is the inner radius of the intestine, which is 0.18 cm [30]; and *l *is the length of the intestine.

### Pharmacokinetic studies

#### Dose proportional oral pharmacokinetic studies

Male SD rats weighing 200-220 g were fasted overnight (12-14 h) before dosing and had free access to water throughout the experimental period. Lumefantrine in 0.25% CMC suspension was administered orally at a dose of 10, 20 & 40 mg/kg to groups of five rats at each dose level. Animals were provided with standard diet 3 h after dosing. The rats were anaesthetized using ether and blood samples (approximately 0.25 mL) were collected from the retro-orbital plexus into heparinized microfuge tubes at 0.5, 2, 5, 8, 24, 30, 48, 54, 72 and 120 h post-dosing. Plasma was harvested by centrifuging the blood at 13000 rpm for 10 min on Sigma 1-15 K (Frankfurt, Germany) and stored frozen at -70 ± 10°C until bioanalysis.

#### Intravenous pharmacokinetic study

Another group of male SD rats (N = 4) weighing 200-220 g, were used in this part of the study. The intravenous formulation was prepared in DMF-PEG 400-ethanol-water (5: 2.5: 1: 1.5 v/v) and finally filtered through 0.22 μm filter before administration. The solution of lumefantrine was administered to rats via a lateral tail vein as a bolus dose of 0.5 mg/kg. Animals had free access to food and water throughout the experimentation period. Blood samples (approximately 0.25 mL) were collected from the retro-orbital plexus into heparinized microfuge tubes at 0.08, 0.5, 2, 4, 6, 25, 30, 48, 54, 72, 96 and 120 h post-dosing and plasma was harvested by centrifuging the blood at 13000 rpm for 10 min and stored frozen at -70 ± 10°C until bioanalysis.

### Sample preparation

A simple liquid-liquid extraction method was followed for extraction of lumefantrine and desbutyl-lumefantrine from rat plasma. To 100 μL of plasma in a tube, 10 μL of IS solution (halofantrine at 1 μg/mL in methanol), 50 μL of GAA, 50 μL of phosphate buffer (50 mM, pH 3) were added and mixed for 15 s on a cyclomixer (Spinix Tarsons, Kolkata, India). Next a 2 mL aliquot of extraction solvent, n-hexane was added. The mixture was then vortexed for 3 min, followed by centrifugation for 5 min at 2000 × g at 20°C on Sigma 3-16 K (Frankfurt, Germany). The organic layer (1.6 mL) was separated and evaporated to dryness under vacuum in speedvac concentrator (Savant Instrument, Farmingdale, USA). The residue was reconstituted in 200 μL of the mobile phase and 10 μL of this solution was subjected to LC-MS/MS analysis.

### LC-MS/MS analysis of lumefantrine and desbutyl-lumefantrine in study samples

Plasma concentrations of lumefantrine and desbutyl-lumefantrine were determined using partially validated LC-MS/MS method that was accurate, precise, specific, sensitive and reproducible. Analyses were carried out using a HPLC system consists of Series 200 pumps and auto sampler with temperature controlled Peltier-tray (Perkin-Elmer instruments, Norwalk, USA) on a XBridge RP18 column (4.6 × 50 mm, 5.0 μm). The system was run in isocratic mode with mobile phase consisting of acetonitrile: methanol (50:50, v/v) and 0.01 M ammonium acetate (pH 4.5) in the ratio of 95:5 (v/v) at a flow rate of 0.65 mL/min. Mass spectrometric detection was performed on an API 4000 mass spectrometer (Applied Biosystems, MDS Sciex Toronto, Canada) equipped with an API electrospray ionization (ESI) source. The mass spectrometer was operated at ESI positive ion mode and detection of the ions was performed in the multiple reaction monitoring (MRM) mode, monitoring transition of m/z 529 precursor ion [M+H]^+ ^to the m/z 511.3 product ion for lumefantrine, m/z 472.1 precursor ion [M+H]^+ ^to the m/z 454.1 product ion for desbutyl lumefantrine and m/z 502 precursor ion [M+H]^+ ^to the m/z 511.3 product ion for IS. Data acquisition and quantitation were performed using analyst software version 1.4.1 (Applied Biosystems, MDS Sciex Toronto, Canada). The retention times for lumefantrine, desbutyl-lumefantrine and IS were 4.81, 2.61 and 2.30 min, respectively. The lower limit of quantification of the method was 2 ng/mL and linearity in the calibration curve standards were demonstrated up to an upper limit of 500 ng/mL. Prior to the analysis of samples, three concentrations (nominal concentrations of 8, 180 and 400 ng/mL) of quality control (QC) samples were prepared in rat plasma. Along with the study samples, QC samples (N = 4, at each concentration level) were distributed among the unknown samples in the analytical run.

### Pharmacokinetic analysis

Plasma data were subjected to non-compartmental pharmacokinetics analysis using WinNonlin (version 5.1, Pharsight Corporation, Mountain View, USA). The observed maximum plasma concentration (C_max_) and the time to reach the maximum plasma concentration (T_max_) were obtained by visual inspection of the experimental data. The area under the plasma concentration time curve (AUC_0-t_) was calculated using linear trapezoidal method. The total area under the plasma concentration-time curve from time zero to time infinity (AUC_0-∞_) was calculated as the sum of AUC_0-t _and C_last_/kel, where, C_last _represents the last quantifiable concentration and Kel represents the terminal phase rate constant. The apparent elimination half-life (t_1/2_) was calculated as 0.693/kel and the kel was estimated by linear regression of the plasma concentrations in the log-linear terminal phase. Clearance (CL) following i.v. dosing was calculated as Dose/AUC_0-∞_. The apparent volume of distribution (Vd) was given by the quotient between CL and elimination rate constant kel following administration of the intravenous bolus dose.

The absolute bioavailability (%F) of lumefantrine was calculated using the relationship,

$$\%\text{F}=\left[\text{AU}{\text{C}}_{\left(0-\infty \right)\;\text{oral}}\times \text{Dose}\left(\text{i}.\text{v}.\right)∕\text{AU}{\text{C}}_{\left(0-\infty \right)\;\text{i}.\text{v}.}\times \text{Dose}\left(\text{oral}\right)\right]\times 100$$

## Results and discussion

### Analytical results

**Figure 1:**
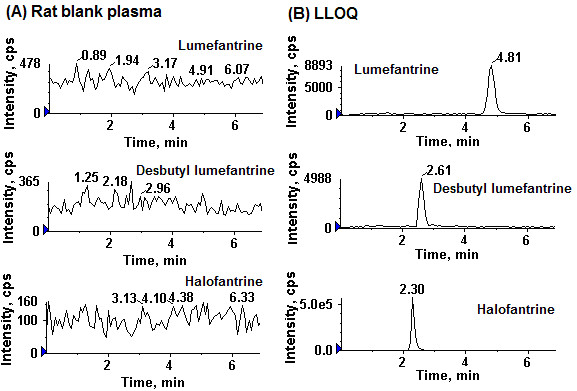
**Typical multiple reaction monitoring chromatograms of lumefantrine and desbutyl lumefantrine in rat plasma (A) Rat blank plasma, (B) drug free plasma spiked with lumefantrine and desbutyl-lumefantrine at LLOQ (2 ng/mL) and halofantrine (IS)**.

The rat plasma samples generated following oral and intravenous administration of lumefantrine were analyzed by the partially validated method along with QC samples. Linearity, specificity & selectivity, recovery, matrix effect and accuracy & precision were measured and used as the parameter to assess the assay performance. The peak area ratios of analytes to internal standard in rat plasma were linear over the concentration range 2-500 ng/ml for both the analytes. The choice of the regression methods was determined. Both lumefantrine and desbutyl-lumefantrine data fit well with a linear regression model, and weighting of 1/concentration^2^. The correlation coefficients of the standard curves for lumefantrine and desbutyl-lumefantrine, ranging from 2 to 500 ng/ml, were all > 0.996. LC-MS/MS analysis of the blank plasma samples showed no interference with the quantification of lumefantrine, desbutyl lumefantrine and IS (Figure-1). The extraction recovery of analytes, was determined by comparing the peak areas of extracted plasma (pre-spiked) standard QC samples (N = 6) to those of the post-spiked standards at equivalent concentrations [31]. The effect of rat plasma constituents over the ionization of analytes and IS was determined by comparing the responses of the post-extracted plasma standard QC samples (N = 6) with the response of analytes from neat standard samples at equivalent concentrations [31]. The recovery and matrix effect testing was performed at three concentrations QC low, QC medium and QC high concentrations viz., 8, 180, and 400 ng/mL for analytes, whereas the recovery and matrix effect of the IS were determined at a single concentration of 50 ng/mL. The extraction recoveries of the lumefantrine and desbutyl-lumefantrine ranged from 70.45 to 80.12%, and the extraction recovery of the internal standard was 73.31%. The ion suppression or enhancement by plasma was less than 12% for the analytes and IS which demonstrated that the matrix effects do not cause quantitation bias.

The intra-day assay precision and accuracy were estimated by analyzing six replicates at four different QC levels, i.e., 2 ng/mL (lower limit of quantitation, LLOQ), 8 (QC low), 180 ng/mL (QC medium) and 400 ng/mL (QC high). The inter-day assay precision was determined by analyzing the four levels QC samples on three different runs. The intra- and inter-day assay precision ranged from 3.74 to 7.63% and 5.79 to 7.32% (R.S.D. %), respectively, and intra- and inter-day assay accuracy were between 95.28 to 105.46% and 96.51 to 105.09%, respectively for both the analytes. The mean predicted concentrations of QC samples (distributed among the unknown samples) were between 89.98-107.56% of the nominal values.

### *In-situ *permeability study

*In-situ *perfusion of intestinal segments of rodents (rats or rabbits) is frequently used to study the permeability and absorption kinetics of drugs. The *P_eff _*values of lumefantrine was determined as the average of six 15 min sampling periods starting from 30 min after the initiation of perfusion, when steady-state had been achieved. Phenol red was used as non-absorbable marker for correction of water flux. During in-house permeability study of amongst USFDA approved high permeability markers, metoprolol showed minimum permeability in rat jejunum (1.88 × 10^-5 ^cm/s). *P_eff _*value of lumefantrine was found to be 4.37 × 10^-5 ^cm/s which is greater than metoprolol permeability. Therefore, lumefantrine can be classified under high permeability class of BCS (biopharmaceutical classification system).

### Pharmacokinetic study

The plasma concentrations of lumefantrine were measurable up to 120 hr after oral and intravenous administration. Figure- 2 depicts the mean plasma concentration-time profiles of lumefantrine following single oral and intravenous administration to male SD rats. The mean oral and intravenous pharmacokinetic parameters for lumefantrine are summarized in Table 2. The variability in plasma concentrations between-animals were observed for lumefantrine after oral administration. However, the low between-animal variability in plasma concentrations after intravenous doses suggests absorption to be critical for between-animal variability in drug exposure. This is also seen in clinical use with substantial inter-individual variability in the pharmacokinetics of lumefantrine after oral administration [14,18].

**Figure 2:**
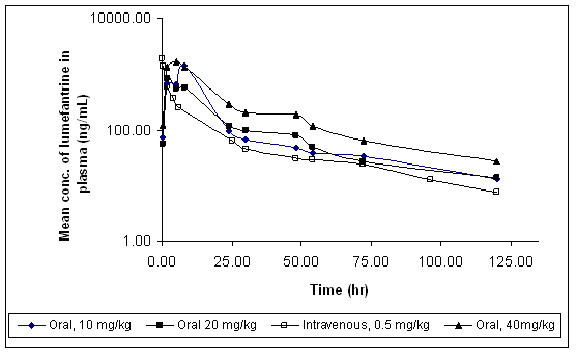
**Plasma concentration versus time profiles of lumefantrine after oral and intravenous administration in rats (N = 5)**. All concentrations are on the logarithmic scale.

**Table 2:** Pharmacokinetic parameters of lumefantrine after oral and intravenous administration in rats

Parameters	Intravenous	Per-oral		
	**0.5 mg/kg**	**10 mg/kg**	**20 mg/kg**	**40 mg/kg**

AUC_0-t _(hr*ng/mL)	9,189.76 ± 1,372.42	21,294.02 ± 3,235.75	17,683.7 ± 3,168.23	38,248.94 ± 7,792.61
AUC_0-∞ _(hr*ng/mL)	9,529.47 ± 1,283.18	22,025.48 ± 3,448.87	18,281.07 ± 3,062.84	39,958.71 ± 8,362.60
**C_max _(ng/mL)**	1,890 ± 330.61	1,488 ± 311.47	938.75 ± 370.64	2,280 ± 522.32
**T_max _(hr)**	-	8(2-8)	3.5(2-8)	5(2-8)
**Vd (L/kg)**	2.40 ± 0.67	-	-	-
**CL (L/h/kg)**	0.03 ± 0.02	-	-	-
**t_1/2 _(hr)**	30.92 ± 4.81	36.08 ± 8.52	25.7 ± 1.85	38.23 ± 4.51
**% F**	-	11.56	4.80	5.24

The T_max _of lumefantrine after oral administration was found to be in the range of 2-8 h. The reason for longer T_max _seems to be the low aqueous solubility of lumefantrine since, lumefantrine displayed high permeability in the *in-situ *permeability study. Similarly in humans the T_max _of lumefantrine occurs later, at approximately six hours post-dosing in healthy volunteers and 3-4 hours in malaria patients [14,18].

For nominal doses increasing in a 1:2:4 proportion, the C_max _and AUC_0-∞ _values increased in the proportions of 1:0.63:1.53 and 1:0.83:1.81, respectively. Both C_max _and AUC_0-∞ _values of lumefantrine were not increased proportionally with increment of dose, which could be due to dissolution-limited absorption at higher doses due to low solubility of lumefantrine.

The plasma concentrations following intravenous administration of lumefantrine dropped to 45% in approximately 0.05 h. Following intravenous administration, the t_1/2 _was found to be 30.92 (± 4.81) h. AUC_0-∞_, clearance (CL) and volume of distribution (Vd) of lumefantrine following administration of 0.5 mg/kg i.v. were 9529.47 (± 1283.18) ng.h/mL, 0.03 (± 0.02) L/h/kg and 2.40 (± 0.67) L/kg, respectively.

The Vd value (2.40 L/kg) of lumefantrine is greater than the total blood volume (0.054 L/kg) indicating extensive extravascular distribution. Furthermore, the mean hepatic blood flow in rats is approximately 3.22 L/h/kg [32]. Using the haematocrit in rat of 0.48 [32], this yields a mean hepatic plasma flow of 1.74 L/h/kg. The CL value for lumefantrine (0.03 L/h/kg) represents less than 2% of the hepatic plasma flow (1.74 L/h/kg), indicating that lumefantrine is low extraction compound. Absolute oral bioavailability (% F) of lumefantrine across the tested doses ranged between 4.80% and 11.56%. The bioavailability was decreased at higher doses. This non-linear relationship between dose and bioavailability is well described for other highly lipophilic drugs, e.g. halofantrine [33]. The variable bioavailability of lumefantrine between individual doses was also observed in humans [18]. The bioavailability of a drug determines the amount reaching the systemic circulation and it in turn determines the pharmacological effects. Hence, preclinical pharmacokinetic data will be of immense help for deciding the partner drug's dose and concentration(s) required for therapeutic efficacy in order to keep the drug's concentration at or above cidal concentration in order to prevent/delay the drug resistance at sub-cidal level.

**Figure 3:**
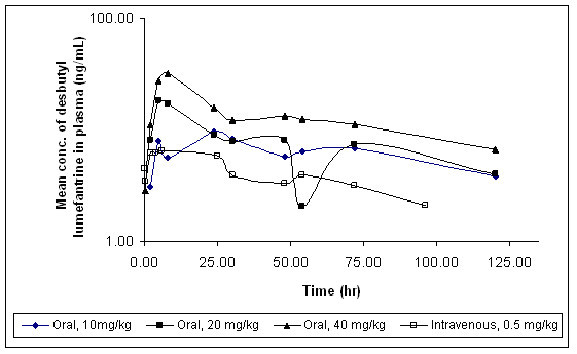
**Plasma concentration versus time profiles of desbutyl-lumefantrine after oral and intravenous administration of lumefantrine in rats (N = 4)**. All concentrations are on the logarithmic scale.

The plasma concentrations of desbutyl-lumefantrine were measurable up to 120 h after oral and up to 96 hr after intravenous administration. Figure 3 depicts the mean plasma concentration-time profiles of desbutyl-lumefantrine following single oral and intravenous administration of lumefantrine to male SD rats. The mean oral and intravenous pharmacokinetic parameters for desbutyl-lumefantrine are summarized in Table 3. The desbutyl-lumefantrine was detected from 2 hr time point, except at 40 mg/kg where it was detected from first time point i.e. 0.05 hr. For nominal doses increasing in a 1:2:4 proportion, the C_max _and AUC_0-t _values increased in the proportions of 1:1.45:2.57 and 1:1.08:1.87, respectively. Following the intravenous administration of lumefantrine, the C_max _and AUC_0-t _value of desbutyl-lumefantrine was found to be 7.91 (± 1.89) ng/mL and 375.75 (± 74.26) ng.h/mL, respectively. In conclusion, we successfully derived the pharmacokinetic parameters of lumefantrine and its metabolite desbutyl-lumefantrine in rats for the first time. Lumefantrine displayed similar pharmacokinetics in the rat as in humans [14,18], with multiphasic disposition, low clearance, and a large volume of distribution resulting in a long terminal elimination half-life.

**Table 3:** Pharmacokinetic parameters of desbutyl-lumefantrine after oral and intravenous administration of lumefantrine in rats

Parameters	Intravenous	Per-oral		
	**0.5 mg/kg**	**10 mg/kg**	**20 mg/kg**	**40 mg/kg**

AUC_0-t _(hr*ng/mL)	375.75 ± 74.26	828.18 ± 281.31	897.39 ± 151.38	1545.89 ± 488.21
**C_max _(ng/mL)**	7.91 ± 1.89	13.54 ± 4.27	19.66 ± 3.72	34.76 ± 17.85
**T_max _(hr)**	13.5 (2-25)	24(5-30)	5(5-8)	8(5-8)

## Competing interests

The authors declare that they have no competing interests.

## Authors' contributions

W and SPS designed and performed the experiments, analysed the data and, wrote the paper. KSRR and AN helped in performing the experiments. SKP and GKJ contributed in review of manuscript. All authors read and approved the final manuscript.
